# ESG assessment methodology for emerging technologies: plasma *versus* conventional technology for ammonia production †

**DOI:** 10.1039/d4su00423j

**Published:** 2024-12-12

**Authors:** Le Yu, Amin Keilani, Nam Nghiep Tran, Marc Escribà-Gelonch, Michael Goodsite, Sukhbir Sandhu, Harpinder Sandhu, Volker Hessel

**Affiliations:** a School of Chemical Engineering, Faculty of Science, Engineering and Technology, The University of Adelaide Adelaide Australia volker.hessel@adelaide.edu.au; b Institute of Process and Particle Engineering, Graz University of Technology Graz Austria; c Institute of Sustainability, Energy and Resources (ISER), The University of Adelaide Adelaide Australia; d DBA Center, University of Lleida Lleida Spain; e Centre for Workplace Excellence (CWeX), University of South Australia, Business, University of South Australia Adelaide Australia; f Ararat Jobs and Technology Precinct, Federation University Australia Ballarat Australia; g School of Engineering, University of Warwick Coventry UK

## Abstract

Environmental, social and governance (ESG) criteria demand that enterprises should not be assessed solely on their financial performance, but also on their environmental, social, and governance performance. This numerical assessment of ESG criteria enables them to be evaluated with the consideration of other financial issues of enterprises' performance and thereby guides financial investments into environmentally and socially responsible firms. ESG, however, solidifies the continuance of conventional technologies but can potentially disadvantage emerging technologies. This study is the first to forecast the ESG potential of emerging chemical technologies. The Morgan Stanley Capital International (MSCI) rating system is applied to one of the top 3 global chemical processes. Ammonia (NH_3_) is produced *via* the Haber–Bosch (HB) process, which needs a huge fossil fuel input and high energy consumption, leading to a significant contribution to carbon dioxide (CO_2_) emissions. In contrast, the ESG assessment rates emerging plasma technology and its spearhead companies that lead innovation and development in this field, which provide the benefits of being a clean, sustainable alternative for green NH_3_ production. Five different plasma-technology companies are considered, with the technology readiness level (TRL) ranging from 3 to 9. These are compared to five different conventional HB companies. We examine the final ESG result of the plasma technology companies, exploring their environmental advances and social viability. In this study, five different themes were selected, including eleven issues, to measure the plasma-technology company's management related to ESG risks and opportunities.

## Abstract 2

This study provides a demonstrated ESG assessment of emerging plasma-technology companies, exploring their potential for environmental advances and social viability, which highlights their sustainability compared to traditional methods.

## Graphical abstract

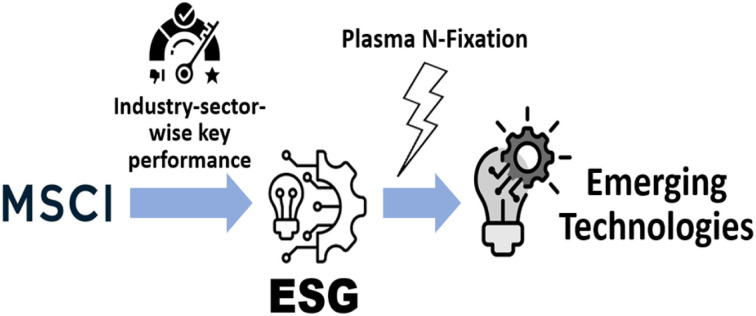

**Sustainability spotlight**

Ammonia (NH_3_) is mainly produced *via* the Haber–Bosch (HB) method, which has a significantly high capital cost and carbon footprint. Plasma-assisted NH_3_ production provides a sustainable alternative to the traditional HB process. Currently, plasma-assisted NH_3_ manufacturing is still under the development stage, and it is of great importance to explore its industrial potential as well as the environmental and social impacts on the commercialization. The adapted MSCI-ESG assessment aims to project the industrial potential specifically for emerging technologies. This proposed ESG assessment has successfully investigated five plasma-assisted companies, which lays the foundation for ESG ratings towards the environmental and social credibility. Our study emphasizes the significance of the UN sustainable development goals: affordable and clean energy (SDG 7), ensure sustainable consumption and production patterns (SDG 12), and climate action (SDG 13).

## 1 Introduction

Chemical manufacturing and fertilizer production enterprises with their sales exposing towards the markets have higher valuations on the sustainability.^1^ Investors consider environmental, social, and governance (ESG) together with sustainability as an important part of their portfolio assessment. While the first encompasses a company's environmental impact, *e.g.* carbon emissions and resource use; social responsibility, *e.g.* employee welfare and community engagement; and governance practices, *e.g.* transparency and ethical leadership, sustainability aligns with long-term value creation, ensuring that investments contribute positively to societal and ecological systems, while mitigating risks associated with climate change and regulatory pressures. Such a holistic approach allows investors to identify resilient and forward-thinking companies that are better positioned for future challenges and opportunities. More investment is being made regarding the sustainable assets, amounting to US$41 trillion by 2022.^2^

Current global ammonia (NH_3_) production ranks as the second most produced chemical globally, with approximately 176 million tonnes per annum (mtpa) worth $80 billion.^3^ Approximately 70% of the synthesized NH_3_ is utilized as an essential precursor towards nitrogenous commodity chemicals in the fertilizer industry.^4,5^ The remainder is used for other industrial applications, such as the production of plastics, polyimides, nitric acid, nylon, and pharmaceuticals.^6^ Over the last century, the importance of NH_3_ production has been extensively acknowledged due to increasing demand in the chemical fertilizer sector.^7^ The global NH_3_ market size is anticipated to grow at a compound annual growth rate (CAGR) of 5.4% by 2030.

More than 90% of NH_3_ is commercially produced *via* the Haber–Bosch (HB) synthesis process, which was developed in 1913.^8^ This conventional process combines hydrogen and nitrogen together with an iron oxide catalyst under a high pressure (200–400 atm) and moderate temperature (400–650 °C).^9^ Over the decades, extensive studies have been undertaken for optimization of the HB process, but the overall energy consumption using fossil fuel remains high, accounting for 2% (8.6 EJ) of the total energy consumption globally.^10^ This energy-intensive process leads to a carbon footprint of 1.5–1.6 kg CO_2e_ per kg NH_3_, due to hydrogen (H_2_) production *via* steam methane reforming (SMR).^11^ Due to its high reactivity, ammonia can significantly contribute to air and water pollution, including eutrophication in aquatic ecosystems and the formation of fine particulate matter (PM2.5) in the atmosphere, both of which pose serious risks to human health and biodiversity. Ammonia can additionally create harmful secondary pollutants, which make its contamination potential approximately four times more severe than that of CO_2_, particularly when referring to global warming potential. Consequently, there is a strong motivation to improve the sustainability level of fossil-based NH_3_ production.

To reduce the environmental impact, enormous efforts have been made to decarbonize NH_3_ synthesis at a commercial scale *via* three major categories: (1) applying carbon capture and sequestration technologies coupled with large HB plants, which is a transitional step for the industry;^11^ (2) replacing H_2_ production *via* SMR (Steam Methane Reforming) with renewable sources; and (3) producing nitrogen (N_2_) through a plasma-based NO_*x*_ synthesis process. Water electrolysis technology is applied to generate green H_2_ using renewable energy (*e.g.*, solar or wind) or biomass gasification.^12,13^ To build a green NH_3_ plant, a distributed production plant at a small scale (known as the mini-HB plant) is recommended for a local market, rather than centralized large-scale manufacturing.^14^ For example, Yara International (Australia) has demonstrated a project to replace fossil-based ammonia plants by introducing renewable H_2_ since 2018. This demonstrated plant aims to supply the first green NH_3_ (20.5 ktpa) to the market with an additional 30% carbon footprint reduction.^15^ However, mini-HB plants still operate under high pressure (100–250 bar) and temperature (350–550 °C).

Alternatively, plasma technology, which allows chemical activation at both high and low temperature, could be another approach to generating green H_2_. Provided that it is accessible to the renewable energy at the sites, plasma processing could be installed ideally for sustainable NH_3_ production. High thermal plasma (HTP) methane (CH_4_) pyrolysis is one of the innovative technologies, where CH_4_ is split into H_2_ and solid carbon with no carbon emission.^16,17^ This process improves the conversion of electrical to chemical energy with a controllable and tuneable heating source, which is suitable for endothermic processes.^18^ To apply the HTP process for cleaner HN_3_ production, Monolith Inc. built the first pilot plant using methane pyrolysis in 2014 at Port Redwood City (California). With the harness of clean electricity, H_2_ is successfully produced at a rate of 20 kg h^−1^ through a thermal plasma-powered pyrolysis of natural gas. By 2020, the commercial scale of H_2_ production (600 kg h^−1^) was completed at Olive Creek (Nebraska), producing 4000 Mt of H_2_ annually along with 13 000 Mt of carbon black as another valuable by-product.^19^

Given that the application of plasma technology towards NH_3_ production is still in the early development stage, it is significant to investigate its industrial potential and determine the social and environmental sustainability of the commercial process. In response to this issue, the application of environmental, social and governance (ESG) for comprehensive sustainability development has gained paramount attention across the global financial markets. The ESG principle is often used as a framework system for responsible investment, defining a strategy to incorporate ESG factors into stakeholder decisions. ESG is therefore set as a standard to measure and reward environmental and social performance along with recognising appropriate governance structures.

Consequently, an organization's ESG score reflects its performance in environmental sustainability, human resource practices, business ethics, and social responsibility. These metrics provide valuable insights for investors, analysts, and other stakeholders to assess risks and opportunities. ESG metrics also guide companies in decision-making to enhance sustainability and ethical practices, enabling benchmarking and comparisons across organizations. Most of the studies show that a high ESG score/disclosure has a favourable impact on companies' performance.^20–22^ Separately, enterprises have taken several contingency actions to cope with the unpredicted ESG/corporate social responsibility (CSR) risks. These actions involve different strategies, such as decreasing waste and carbon emissions, applying clean and sustainable energy, producing green products, collaborating with sustainable supply-chain companies, improving employees' well-being with a safe working environment, and respecting employees' human rights. Regarding environmental change commitments, these key changes could also improve the ratings of their ESG indicators, inevitably having a particular level of contribution to their profitability. Nevertheless, maintaining the flow of information enables decision-makers to ensure whether such actions might lead to higher operating costs and reduced profits or returns on assets (ROA).

It has been proven that there is a nonlinear U-shaped and positive relationship between ESG indicators and the temporary financial performance of the aerospace industry.^20^ Moreover, ESG performance is considered as one of the essential measurement standards and indicators of CSR for the development of sustainability. In this study, we adapt recent findings on assessment of ESG performance towards manufacturing enterprises. To achieve this, ESG indicators with their fiscal information across 100 worldwide enterprises from 2005 to 2020 were collected to build a multilevel quadratic growth model. This established model was then applied to investigate the impact of ownership structures from the different industries and disclosed information on ESG/CSR risks and opportunities. The key finding can be a practical reference for strategy formulation to manage CSR risks and seek opportunities related to the improvement of companies' ESG performance.

This study aims to help emerging technologies, especially plasma technology for NH_3_ production, as these are needed to solve our urgent economic and environmental challenges. The ESG rating intrinsically disadvantages emerging technologies, as it is organised to reflect industrial maturity. There is a belief that ESG ratings suffer from the “quantity bias effect” (OECD, 2021), meaning a relationship between the size and disclosed resources of a company and the availability of a company's sustainability (ESG) performance.^23^ This has been stated for MSCI's ESG ratings.^24^ A study used Thomson Reuters ASSET4 ESG ratings for a thorough investigation of ESG scores.^25^

This study aims to correct that bias and disadvantage, by proposing to project the potential of industrial maturity of emerging technologies in the near future (*e.g.*, five years), which may help to translate their potential to reality. Evidently, currently used ESG parameters may not entirely be suited for this ‘*ex ante*’ ESG analysis. This study needs not only to exclude and substitute ESG parameters, but also to improve the scientific credibility and traceability to correct bias. Critically, it needs to be surveyed what the loss of accuracy is from those changes and how meaningful such analysis is. Finally, the proposed methodology has been applied to assess the ESG readiness of five emerging plasma technology companies *versus* five conventional HB companies. This study aims to lay the foundation for an ESG score methodology to measure corporate sustainability and to inform sustainable and responsible investors to make decisions based on the ESG score.

## 2 Methodology

### 2.1 Commercial ESG standards

ESG scores are evaluated using methodologies with a combination of company disclosures, news, and public information including proprietary data.^26^ Industry related ESG scores involve machine learning and human validation that is done by experts with extensive experience in evaluating companies, industries, and ESG-related topics.^26^ Low consensus about the definition of ESG scores exists among academics. This study uses three widely impactful commercial ESG ratings that have demonstrated commercial and societal impact, *e.g.*, in collaboration with the stock exchange (Dow Jones) and the United Nations.

#### 2.1.1 S&P 500 ESG Elite Index

The S&P 500 ESG Elite Index values sustainability and ethical business practices, using best-in-class criteria; approximately 20% in the S&P 500 (The Standard and Poor's 500, S&P 500). The ESG Elite Scores are based on the S&P Global ESG Scores *via* the S&P Global Corporate Sustainability Assessment (CSA). The latter is a questionnaire-based analysis process that aims to identify the extent to which companies are ready to apprehend and respond to upcoming sustainability opportunities and challenges in the global market. It is used as a benchmark for ethical and sustainable investing, by providing investors with exposure to companies that align with global sustainability goals, such as reduced carbon emissions and social responsibility, without sacrificing market returns.

#### 2.1.2 Sustainalytics

Sustainalytics is defined as an enterprise that evaluates the sustainability performance of selected manufacturing companies based on their environmental, social, and corporate governance (ESG) analysis.^27^ Its ESG rating system incorporates a company that is exposed to industry-specific risks and its ability of risk management.^28^

The Sustainalytics rating is a two-step process, based on risk exposure and management. The exposure describes the degree of vulnerability to general material ESG risks and material ESG issues (MEI) level. The exposure scores of MEI are first evaluated at the subindustry level and then improved at the company level. The management response of a company is divided into two parts: (1) the manageable risk and (2) unmanageable risk, which is provided by a manageable risk factor (MRF). Again, at a subindustry level, a pre-assessment is made. The range of MRFs covers between 30% and 100%, amounting to the risk exposure deemed to be manageable by a company.

Since 2018, Yahoo! Finance has included Sustainalytics' ESG scores across over 2000 companies.^29^ In 2013, Sustainalytics together with the United Nation's Global Compact launched the Global Compact 100 index, providing an up-to-date stock index to track Global Compact signatories.^30,31^ Five years later, the World Bank published a sustainable development note related to Sustainalytics' Global Sustainability Signatories Index, providing an alternative way to track Global Compact signatories with an improved sustainability rating system.^32^

#### 2.1.3 Morgan Stanley Capital Investment and Global Reporting Initiative

Morgan Stanley Capital Investment, MSCI Inc., is a US finance company and a global provider of services, including real estate indices, stock indices, portfolio risk and performance analysis tools, and ESG.^33^ It involves the MSCI World, MSCI All Country, World Index (ACWI) and MSCI Emerging Markets Indices.^33^ With the highest score of 10, the Governance Pillar Score is set as the sum of deductions generated from Key Metrics of Corporate Governance.

The Global Reporting Initiative (GRI) is defined as an international standards organization that facilitates governments, businesses, and other organizations to understand their impacts on issues such as climate impact, human rights, and economy.^34^ The founders of GRI are the United Nations Environment Programme, Ceres, and the Tellus Institute. MSCI ratings are based on GRI standards.^24^

### 2.2 MSCI-GRI ESG scoring and adaptation used in this study

#### 2.2.1 E&S focus of MSCI-GRI

This study chooses the MSCI score methodology based on GRI definitions, for reasons as reported in the Results and discussion section. The study was conducted using two key ESG pillars (environmental and social, E&S) for 5 plasma-based and 5 conventional technologies. The pillar of ‘governance’ had to be excluded, as governance indicators are not always publicly available and/or deducible especially for firms in emerging plasma technologies. Furthermore, while innovative governance models have been qualitatively reported for emerging technology companies, there is no direct and quantifiable correlation between the establishment of a specific technology and ways of governance.^35^ Therefore, to ensure comparability between conventional and plasma technologies we focussed on environmental and social indicators. However, because the term ESG has widespread recognition and acceptance within the literature and practice (as compared to ES), we continue to adopt the umbrella term ESG in this research (despite focussing only on E and S).

#### 2.2.2 Theme selection within MSCI by GICS

The Global Industry Classification Standard (GICS) is a comprehensive framework designed to categorize companies worldwide based on their primary business activities with uniform and detailed industry definitions. Using GICS, the industrial category ‘15101030 Fertilizers & Agricultural Chemicals' was chosen under ‘151010 Chemicals’ from the industrial sector ‘15 Materials'. The themes and issues from GICS are set based on the specific industrial sectors such as chemistry-fertilizers.

#### 2.2.3 Theme and issue exclusion or adaptation

MSCI-GRI scores for E&S are given along ‘Themes,’ which were broken down into several ‘Issues,’ providing a scientific definition for a quantitative rating. As E-themes (Table S1†), climate change, pollution, and sustainability were considered, while the issue of ‘natural capital’ was excluded. Despite examining several company websites, we were not able to find appropriate natural capital data for emerging companies such as for the theme's issues of biodiversity & land use, raw material sourcing, and water stress. These issues monitor long-ranging effects, demanding the use of technology for an extended time while emerging technology companies have been in existence for shorter periods. As S-themes (Table S11†), chemical safety and health & safety were considered. The issue of ‘stakeholder opposition’ was excluded, again for the reason of recency and smaller size of emerging companies.^36^

Some issues were slightly redefined and sharpened to give a better match to the plasma technology. The issue ‘Opportunity in Clean Tech’ (clean technologies) was redefined as ‘Opportunity in renewable energy,’ and ‘Toxic emissions & waste’ was modified to ‘Toxic waste.’ Under the S-themes, human capital and product liability were considered as issues.

#### 2.2.4 Considered themes, their weighing, and issues

**Fig. 1:**
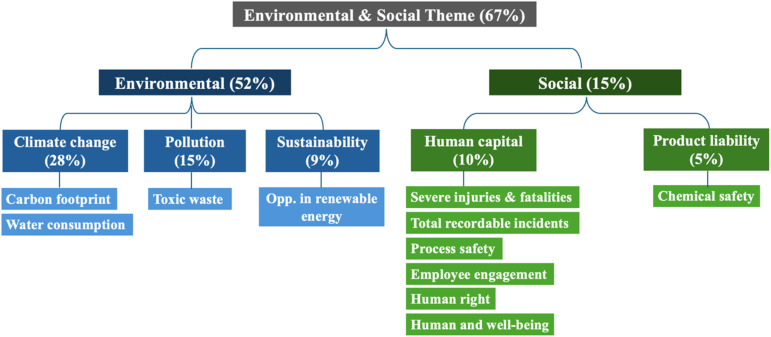
Diagram of E&S selection criteria applied in this study.

The environmental and social (E&S) impacts of emerging plasma-technology companies were evaluated across twelve issues (Fig. 1). By adapting the Global Industry Classification Standard (GICS), climate change (28%) and human capital (10%) were assigned the highest weighting among the environmental (52%) and social pillars (15%) for the fertiliser and agriculture chemicals sector, respectively. It is important to note that the governance pillar was excluded in this study, contributing 33% of the total weight. The overall score of E&S assessment was calculated using eqn (1):^37^

1 Overall E&S score = (*E*_score_ × *W*_E_) + (*S*_score_ × *W*_S_)

where *E*_score_, *S*_score_, and *G*_score_ are the scores of environmental, social and governance themes, respectively. *W*_E_, *W*_S_, and *W*_G_ are the weighting percentages assigned to each theme.

To evaluate the E&S performance of a company, the scoring system was established with the consideration of the risk exposure and its management strategies. Risk exposure was scored from 2–10, where 2 represented no/lowest exposure and 10 represented a very high level of exposure. The risk exposure criteria of the E pillar were created including carbon footprint, water recycling, total solid waste and renewable source applied (Table S1†). The carbon footprint can be expressed as the carbon intensity during NH_3_ production, which was evaluated using eqn (2):^38^

2 Carbon intensity = total CO_2e_ emission (kg)/total product output (tonne)

The water cycling rate indicated the improved efficiency of wastewater reduction, which can be evaluated using eqn (3):^38^

3 Water recycling rate (%) = (quantity of water recycled/total water used) × 100

Based on the information collected from the company, the risk exposure level of each company has been scored in Table S2.† Risk management needs to be commensurate related to the level of exposure. Mitigation actions together with targets were two main categories evaluated within the management criteria for carbon footprint, water consumption and toxic waste issues (Tables S3, S5, S7 and S8†), whereas the management strategy, initiatives and performance were included for renewable energy issues (Tables S9 and S10†). The risk exposure criteria of the S pillar included occupational safety, human capital development and product liability. The potential severe injury & fatality (PSIF) rate, total recordable incidents (TRI) and process safety (PS) index (Table S11†) were selected to provide the score of occupational safety issues. Employment engagement, human rights, and health & well-being (Table S12†) were three main contributors to human development issues. Chemical safety (Table S13†) was the only factor considered for the product liability of the company. Strategies with targets were two main categories contributing to the management criteria of each S issue. The total E&S score was calculated by the combination of E and S scores multiplying their respective weights.

In this study, five plasma companies (1–5) were selected with the TRL range of 3–9, including (Company 1) non-thermal plasma (NTP)-technology company (TRL 6); (Company 2) thermal plasma (TP)-technology company (TRL 9); (Company 3) thermal plasma-technology company (TRL 3); (Company 4) non-thermal plasma-technology company (TRL 3); and (Company 5) non-thermal plasma-technology company (TRL 3). The five conventional companies were used as the benchmarks for further comparison, including two large-scale NH_3_ companies (Companies 6 and 7); two medium-scale NH_3_ companies (Companies 8 and 9); and a small-scale NH_3_ company (Company 10). Due to confidentiality, objectivity and ethical considerations, the real names of the companies were not listed in the study.

#### 2.2.5 Potential limitations and uncertainties

The key limitation of ESG assessment is the lack of standardized rating systems, where different rating agencies, such as Sustainalytics and Bloomberg, utilize various methodologies for ESG score calculation, leading to inconsistency in the measurement of ESG indicators.^39^ Moreover, ESG sources obtained from different company reports may be disclosed inconsistently across regions, leading to data quality issues.^40^ In this study, the companies with a higher TRL (>7) may receive more scrutiny from investment analysis and social media, compared to the ones with a lower TRL. The larger companies also have adequate resources (*e.g.*, sustainability reports) to address the ESG issues, contributing to higher ESG scores.

## 3 Results and discussion

### 3.1 Choice in commercial methodology and its adaptation

An initial decision for this study was how to rate emerging technologies. One way is to develop and refine a new methodology. This can provide scientific insight into a particular aspect of ESG, yet it is unlikely to be holistic or easily transferable. Rather, as another way, this study wants to view plasma as emerging technology through a commercial ESG lens. It is essential that these commercial ESG ratings are detailed in criteria that the medium-TRL (technology readiness level) practice of emerging-technology companies can address, especially relating to the social pillar of ESG.

Three commercial ESG indices are mainly applied for ESG ratings of companies: the S&P 500 ESG Elite Index, Sustainalytics, and the Morgan-Stanley-Capital-Investment (MSCI) of the Global-Reporting-Initiative (GRI).

**Fig. 2:**
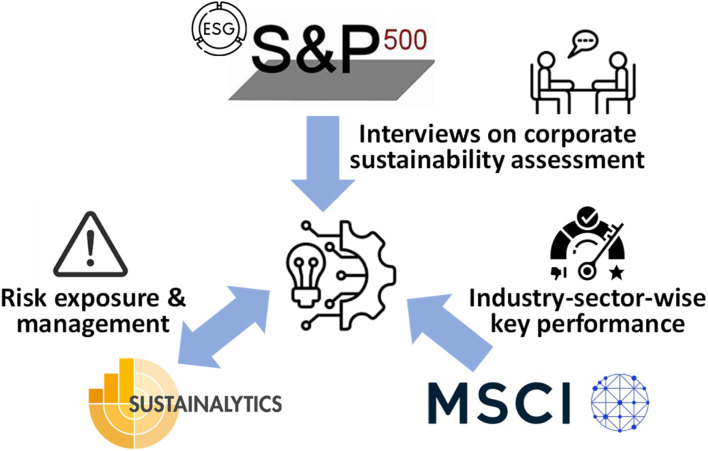
Three commercial ESG methodologies and their way of ESG assessment. In the centre, the icon stands for emerging technology companies, plasma in the context of this study.

The S&P 500 ESG Elite Index (see Section 2.1.1) is closely related to the market and business (similar to the Dow Jones index). This commercial ESG tool was not considered as good a fit for the ESG rating of emerging technologies, as it is done in a weighted interview style, which necessitates large reporting evidence to rate the interview outcome (Fig. 2). (Small) Emerging technology companies have limited degree reporting as compared to large established companies.

Sustainalytics (see Section 2.1.2) provides an approach that may be used for the ESG rating of emerging technologies. Yet, it is entirely based on a ‘receiving mode’ by analysing the degree of external risks for a company and their management, Fig. 2, while in the MSCI-GRC approach used here (see below), risk management is only a part of it. The technology seen here is in a kind of ‘static mode.’

Emerging technology companies undergo fast change and improvement of their new technologies; these are in a ‘sending mode.’ Here, the internal technology position is key with its performance characteristics and ESG-documented evidence (Fig. 2). The MSCI-GRI approach (see Section 2.1.3) suits this kind of assessment *via* key-performance criteria of the technology. It is detailed in its environmental criteria, which are broken down hierarchically into parameters that can be filled in by theoretically derived performance values. The GRI 305 class, for example, addresses emissions into air, which is close to the impact categories of life-cycle assessment, LCA, such as global warming potential or ozone depletion potential. LCA is a common sustainability tool at the academic–industry (TRL 3–6) translation. Many emerging-technology companies are start-ups founded by academics of universities, being ready for delivery of those scientific inputs. The social criteria of MSCI-GRI suit the company reports of those kinds of (larger) emerging-technology companies, which have transitioned from a start-up to a real company, noting that emerging companies are necessarily disadvantaged here as compared to global established companies. For this reason, this study decided to follow MSCI-ESG reporting and modify it for plasma emerging-technology companies.

### 3.2 ‘Helicopter-view’ E&S assessment: five plasma and five conventional companies

This study starts with a ‘helicopter view’ that is then broken down into details. The first question is whether emerging plasma companies, in general, have the potential of an E&S rating similar to established conventional HB companies. We computed the E&S rating of five global plasma companies against five conventional HB companies producing ammonia. The analysis determined that plasma technology provides E&S-documented advances in the environmental pillar. Scientific literature has proven proper use of renewable energy or non-fossil resources, with life-cycle assessment (LCA) quantifying this towards LCA impact categories, including the global warming potential (climate change).^41^ Yet, E&S ‘documentation’ should go one step further, meaning that the plasma companies testify to having developed pilots that meet favourable sustainability criteria. This also means that the theoretical potential of the plasma technology (laboratory-scale for science) has translated into ESG-relevant company reporting.

The social pillar rating of the emerging plasma technology companies is almost as good as for the conventional companies (Table 1). This demonstrates that the plasma technology companies have moved out of tech-focused start-ups to self-determined entities, which start to fulfil standards of global sustainable companies in terms of ESG. Accordingly, the total (combined E&S) score proves that the plasma technology companies have the potential to attract investors.

**Table 1:** Total environmental and social scores for plasma-technology companies (1–5) and conventional HB companies (6–10)^a^

	1	2	3	4	5	6	7	8	9	10
Social pillar	1.2	1.2	1.1	1.1	1.1	1.4	1.3	1.2	1.1	1.0
Environmental pillar	4.7	4.8	4.5	4.3	4.4	3.9	3.7	4.1	3.4	3.7
Total score	**5.9**	***6.0***	**5.6**	**5.4**	**5.5**	**5.3**	**5.0**	**5.3**	**4.5**	**4.7**

a Total E&S score is 6.7. The details of environmental scores for plasma-technology companies (5) and conventional HB companies (6–10) (the highest score is marked in italic font).

The above-computed outcome shows that the social assessment of plasma technology companies is the determining factor for their overall E&S rating, which is logical as this is a soft point. Emerging companies have just entered commercial practice and are intrinsically inferior in social practices. In turn, this also means that social issues define a matter of improvement in total ESG ratings. Some ‘issues’ of the social assessment shall be discussed in detail.

The score of the plasma companies for three safety-related issues compares on average to the five conventional HB companies (Table 2). Both emerging plasma and conventional HB technologies show a large spread, demonstrating that the individuality of companies determines the score rather than their technology affiliation. Concerning the three-fold issues of human leadership, rights and health, the emerging plasma technologies score lower than the five conventional technologies on average. This is not surprising, as those kinds of social issues are expected to be higher with a longer market presence; it is noted that the latter show a large spread. For the issue of chemical compliance and safety, emerging plasma technologies and conventional HB technologies score similarly on average.

**Table 2:** Environmental (top) and social (bottom) scores for plasma-technology companies (1–5) and conventional HB companies (6–10)^a^^,^^b^

Themes	Weighing (%)	Issues	Weighing (%)	1	2	3	4	5	6	7	8	9	10
Climate impact	28	Carbon footprint	14.5	9.6	9.7	9.4	8.7	9.7	7.1	6.9	7.9	6.7	7.3
		Water consumption	13.5	9	9.4	8.6	8.1	8.1	7.8	7.2	8	6.1	7.1
Pollution	15	Toxic waste	15	8.5	8.2	7.5	7.5	7.5	7.4	6.3	7.5	6.6	6.9
Sustainability	9	Opp. in renewable energy	9	9.2	9.5	9.1	8.9	9.1	8.1	8.7	8.2	6.5	7.4
**Total score**				**4.7**	***4.8***	**4.5**	**4.3**	**4.4**	**3.9**	**3.7**	**4.1**	**3.4**	**3.7**
Human capital	10	Severe injuries & fatalities	5	8	8.5	8	7.2	7.2	9	9.8	8.2	9.2	6.8
		Total recordable incidents											
		Process safety											
		Employee engagement	5	7.2	7.2	6.9	6.9	6.9	9.5	7.3	8.9	8.1	7.1
		Human rights											
		Health and well-being											
Product liability	5	Chemical safety	5	8	8	7.5	7.5	7.5	8.8	8.1	7.6	6.8	7.0
**Total score**				**1.2**	**1.2**	**1.1**	**1.1**	**1.1**	***1.4***	**1.3**	**1.2**	**1.1**	**1.0**

a Weighting 52% towards the total ESG score, with the total score of environmental being 5.2 (the highest score is marked in italic font).

b Weighting 15% towards the total ESG score, with the total score of environmental being 1.5 (the highest score is marked in italic font).

### 3.3 Environmental pillar analysis: five plasma companies

With the impetus of the ‘helicopter-ESG’ providing a positive outlook for plasma technology, Table 2 presents the ESG analysis for the environmental pillar towards five different plasma-technology ammonia companies, ranging from TP and NTP companies with TRLs 3–9. Company 2 (HTP-technology with TRL 9) was ranked first with the highest score of 4.8 (out of 5.2), followed by company 1 (NTP-technology with TRL 6) with a score of 4.7. This demonstrates that technological maturity (high TRL) is the key to a high environmental ESG score.

Considering the themes within the environmental pillar, the climate impact occupies the highest weight of 28%, containing two issues of carbon footprint (14.5%) and water consumption (13.5%) (Table 3). This indicates that the climate change score is the most critical issue for evaluating the environmental performance, whereas the sustainability (9%) score appears to be the least determining factor.

**Table 3:** Environmental scores for plasma-technology companies^a^

Themes	Issues	Weighing (%)	1	2	3	4	5
Climate impact	Carbon footprint	14.5	*9.7*	9.6	9.4	8.7	9.7
	Water consumption	13.5	*9.4*	9	8.6	8.1	8.1
Pollution	Toxic waste	15	8.2	*8.5*	7.5	7.5	7.5
Sustainability	Opportunity in renewable energy	9	*9.5*	9.2	9.1	8.9	9.1
**Total score**		**5.2**	**4.7**	***4.8***	**4.5**	**4.3**	**4.4**

a The highest score is marked in italic font.

Carbon footprint is one of the crucial issues in response to numerous effects on global warming, as the NH_3_ industry sector has a significant contribution to greenhouse gas emissions. To evaluate the exposure score of carbon footprint (Table S3 in the ESI†), the carbon intensity for each company was applied and calculated using direct CO_2_ emissions from the production of NH_3_ (tonne CO_2e_ per tonne NH_3_). While carbon intensity refers to the emissions per tonne NH_3_, carbon footprint refers to the total emissions from all sources of CO_2_ emissions involved in the process, consequently making carbon footprint a broader concept, while carbon intensity is a specific measurement that helps quantify and reduce the overall footprint. By using a renewable electricity supply, company 1 based on NTP technology emitted 0.15 tonne CO_2e_ per tonne NH_3_ produced, while company 2 using TP technology generated slightly less CO_2_ (0.13 tonne CO_2e_ per tonne NH_3_ produced). The risk exposure for companies 1 and 2 scored the same as 3 out of 10, as the carbon intensity was less than 1 tonne CO_2e_ per tonne NH_3_. Moreover, companies 3–5 share the same score (2 out of 10), as their NTP technology development is still in the proof-of-concept stage, leading to the least contribution towards carbon emissions. The current technology (HB process) for NH_3_ production requires a steady supply of distilled water in a high volume for the operation, leading to high CO_2_ emissions.^6^ Sustainable NH_3_ production has been explored using water electrolysis coupled with renewable energies (*e.g.*, wind and solar) for H_2_ production. However, water electrolysers have a high demand for pre-treated water with a high purity level.^42^ Plasma-assisted NH_3_ synthesis by activation of H_2_ and N_2_ has been used to tackle some of the major problems associated with the HB process, including low water consumption and low energy requirement for operation.^43^ Compared to the conventional HB process, the majority of plasma-based companies are still in either the research or the development stage.

There are two types of waste during the process, which are non-toxic (non-hazardous) and toxic (hazardous) waste. Non-hazardous waste is commonly from municipal solid waste, such as construction and demolition materials. Hazardous waste is considered harmful to the environment or human health when improper disposal and storage occur. Typical toxic wastes from HB-based NH_3_ manufacturing are catalysts (iron oxide) removed and replaced during the process, chemical residues, and other wastes from the maintenance activities. However, the plasma-based process has its advantages of wide feasibility, low cost and waste, flexibility, and low energy consumption, especially NTP-assisted NH_3_ production.

Table 3 shows that company 2 has the highest score (8.5) under the toxic waste issue, followed by company 1 (8.2), and the remaining three companies share the same value (7.5). Company 2 as one of the low-cost producers of H_2_ has unlocked significant value from carbon sequestration. Its innovative HTP process harnesses the clean electricity for CH_4_ pyrolysis to produce NH_3_ and carbon black (automotive application). This company has demonstrated the reactor technology at a commercial scale, aiming to develop a scalable process. With the highest risk management score, the strategy of the waste reduction has been created to control the final product quality, and a multidisciplinary team is required to optimize the reactor design for environmental management. On the other hand, company 1 developed an advanced NTP-assisted electrochemical process for NH_3_ production with only air and water consumption. However, the main disadvantage of this process is the intermediate product (NO_*x*_) generated from N_2_ activation, posing a harmful impact on the atmospheric environment. NO_*x*_ (nitrogen oxides) contributes to air pollution, acid rain, and the formation of ground-level ozone, which can negatively impact human health and ecosystems. Additionally, NO_*x*_ emissions from industrial processes, such as ammonia production, increase global warming due to their interaction in the formation of secondary pollutants like particulate matter (PM2.5). To manage this risk, a waste absorption system has been set up to reduce the hazardous impact. At the same time, a series of deployments have been involved in an industrial setting to reduce large-scale combustion, including decentralized plant and supply chain removal.

Opportunities in renewable energy are among the principal issues on which companies are evaluated based on their positioning to meet the market demand for renewable power through capacity additions and network expansion (“MSCI ESG Ratings Methodology: Opportunities in Renewable Energy Key Issue”). All companies appear to have the highest performance for the use of renewable energy, providing potential for replacing the conventional process with plasma technology. For example, company 1 has deployed the synthesis modular at any scale, which is compatible with variable renewable electricity supply. Company 2 uses 100% renewable electricity to convert renewable biogas into H_2_ and carbon black. This process provides no scope 1 CO_2_ emissions and significantly reduces life-cycle emissions.

### 3.4 Carbon footprint and risk management: five plasma companies

Table 4 shows the risk management criteria and issue scores for TP and NTP-technology companies. It is important to note that all plasma-based companies have made a significant effort on the use of clean energy and improvement of operational efficiency for energy reduction, aiming to achieve the targeted CO_2_ reduction. Company 1 leads the development by having superior ESG-reporting for ‘Clean sources of energy’ and ‘Reduction of future energy consumption’. Concerning the latter companies, 2 and 5 also have advanced ESG-reporting. Overall, companies 1, 2, and 5 end with a high ESG score for risk management for the carbon footprint issue.

**Table 4:** Risk management score for the carbon footprint issue^a^

Criteria	Score				
	Company 1	Company 2	Company 3	Company 4	Company 5
Clean sources of energy	*1.3*	0.9	1	0.5	0.9
GHG (greenhouse gas) capture plan	0.5	0.5	0.5	0.5	0.5
Energy management and operational efficiency improvement	0.5	0.9	0.9	0.5	0.9
Reduction of future energy consumption	*0.9*	*0.9*	0.5	0.5	*0.9*
Carbon or energy efficiency improvement	0.5	0.5	0.5	0.5	0.5
Demonstrated track record of achieving targets	0.5	0.5	0.5	0.5	0.5
GHG emission reduction plan	0.5	0.5	0.5	0.5	0.5
**Total score**	***4.7***	**4.6**	**4.4**	**3.5**	***4.7***

a The highest score is marked in italic font.

Companies 1 (TRL 6), 2 (TRL 9), and 5 with relatively high values (4.7, 4.6, and 4.7, respectively) of risk management scores have made a significant effort in energy efficiency and process conversion rate, leading to reduced water consumption for operation (Table 2). However, all five plasma-based companies have no established implementation strategy for water reduction for 2024 and beyond. Moreover, responsible water management needs to be promoted and engaged in collaborative efforts with stakeholders, continuing the contribution towards the water-efficient process. With the consideration of further development of the plasma-based company, it is highly recommended to establish an annual track-record for achieving the targeted water reduction with an improved energy-efficiency process.

### 3.5 Social pillar analysis: five plasma companies

Company 1 with the highest TRL score has the best social pillar rating (Table 5). This shows that social pillar reporting is related to TRL achievement. On the positive side, all emerging-technology companies reported well to allow judgment according to the MECI-GRI methodology. ‘Human capital’ can be ranked high in lower TRL companies, as in company 2. ‘Incident Reporting Process Safety…’ and ‘Chemical Safety’ are highest in high TRL companies.

**Table 5:** Social scores for plasma-technology companies^a^

Themes	Issues	Weighing (%)	1	2	3	4	5
Human capital	PSIF	5	8	*8.5*	8	7.2	7.2
	TRI						
	PS						
	Employee engagement	5	*7.2*	*7.2*	6.9	6.9	6.9
	Human rights						
	Health & well-being						
Product liability	Chemical safety	5	*8*	*8*	7.5	7.5	7.5
**Total score**		**1.5**	***1.4***	**1.3**	**1.1**	**1.1**	**1.1**

a The highest score is marked in italic font.

Emerging technologies show promise for better jobs and are better also in terms of occupational safety. Therefore, this issue of the social score, as given in Table 4, is analysed in detail. Other social score issues are listed and quantified in the ESI.†

Plasma company 2 scores highest for the risk management score for occupational safety (Table 6). This is due to their leadership in two criteria: the percentage of the company's H&S system certified to OHSAS 18001 or ISO 45001 (above 20%) and the implementation strategy to achieve targets. Companies 1 and 3 have similarly high scores for the first but fail in the second. Companies 4 and 5 score low for the two criteria. The score in all other criteria is the same for all plasma companies 1–5. The total score of company 2 is notably higher than for 1 and 3, with an even larger gap in the total score between it and companies 4 and 5.

**Table 6:** Risk management score for occupational safety^a^

Criteria	Score				
	Company 1	Company 2	Company 3	Company 4	Company 5
Group-wide H&S policy has been established	0.5	0.5	0.5	0.5	0.5
H&S policy has applied to contractors with a regular audit	0.5	0.5	0.5	0.5	0.5
Percentage of company's H&S system certified to OHSAS 18001 or ISO 45001 (above 20%)	0.9	*1*	0.9	0.5	0.5
Executive body is responsible for the H&S strategy and performance	0.9	0.9	0.9	0.5	0.5
H&S targets cover the target year, reduction (%) and baseline	0.5	0.5	0.5	0.5	0.5
Implementation strategy to achieve targets	0.5	*0.9*	0.5	0.5	0.5
Demonstrated track record of achieving targets	0.5	0.5	0.5	0.5	0.5
H&S metrics include the lost time incident rate, TRI rate and fatalities	0.5	0.5	0.5	0.5	0.5
**Total score**	**4.8**	***5.3***	**4.8**	**4**	**4**

a The highest score is marked in italic font.

### 3.6 ES overview: five plasma companies and one conventional (HB) company

**Fig. 3:**
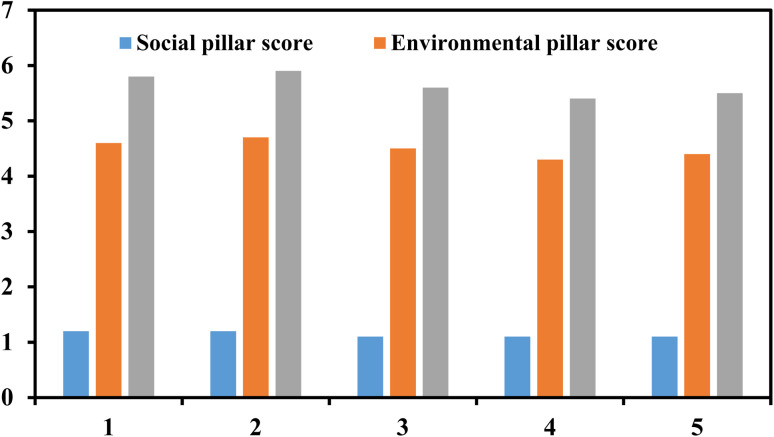
Total score (environmental and social pillar) for five plasma-technology companies.

An overview of the total ES score shows the prevalence of the environmental pillar, which is high for all five plasma companies (Fig. 3). The first two companies with a higher TRL level score higher, yet the difference is small compared to companies 3–5. As given above, all five plasma companies have well-established social reporting. Consequently, the total ES score is relatively similar for the five companies, indicating the best value being 6.0 (company 2) out of a maximum of 6.7.

**Fig. 4:**
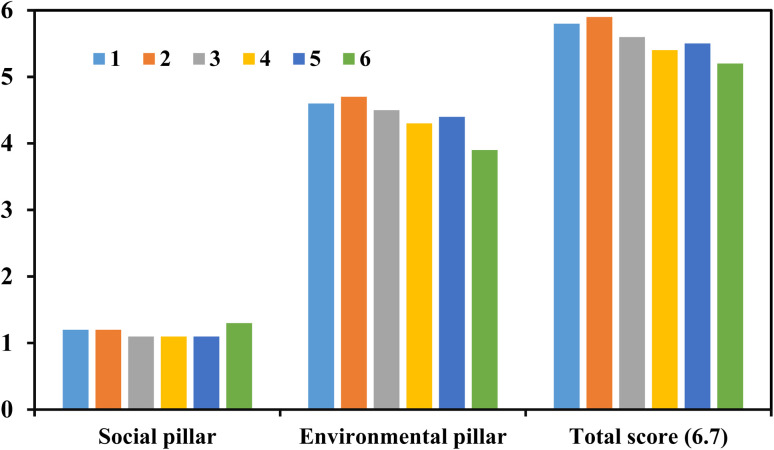
Environmental and social score of five plasma-technology companies compared to a large-scale company. Green bar is a conventional company producing NH_3_ at the largest global scale.

Compared to a commercial HB company producing NH_3_ (Company 6), the plasma companies are a bit behind in the social pillar, which is overcompensated by their improved environmental pillar (Fig. 4). The total score of the five plasma companies is better than that for the commercial HB company. At this point and for clarity and fairness of our conclusions, it must be critically noted that only plasma companies 1 and 2 demonstrated technology efforts that are publicly accessible, while the other companies report based on internal achievements that cannot be publicly checked. It needs to be also noted that the HB company 6 produces at a global scale of several 100 000 t/a NH_3_, while company 1 produces hydrogen at a very few 10 000 t/a H_2_, equivalent to about 40 000–60 000 t/a NH_3_. Company 2 does not report on their nitration fixation capacity, yet it is not estimated to exceed a few t/a NH_3_ or N-equivalent (nitrate).

## 4 Conclusions

This is, to the best of our knowledge, the first study to use commercial ESG ratings for predicting the market potential of emerging chemical and fertiliser companies in the future, in an ‘*ex ante* fashion’ employing Consequential Life Cycle Assessment (CLCA). Plasma technology has recently made a major move towards chemical fertiliser production, yet it has not reached market maturity (with exceptions such as the use of thermal plasmas). This study addresses whether it is sufficient to provide a reliable Environmental and Social (E&S) rating that can be trusted by investors. This study motivates plasma and other emerging technology studies to consider ESG documentation as an asset. It has also demonstrated a methodology to rate emerging technologies, knowing their (intrinsic) deficiencies in full-scale ESG documentation, which adds the ‘*ex ante* viewpoint’ that CLCA has successfully taken.

The results showed that a plasma company with TRL 9 had the highest score (6.0 out of 6.7) for both E (4.8 out of 5.2) and S (1.2 out of 1.5) pillars, indicating also that a higher TRL is effective in promoting risk management and opportunity creation. For the E pillar, the plasma companies with a lower TRL (3) had limited capacity for toxic waste and water consumption management, with no established track records for hazardous impact plans. For the S pillar, plasma companies showed lower risk exposure for occupational safety and product liability due to the clean and safe plasma-assisted process. Yet, the assessment presented lower performance on human capital development with less stakeholder engagement including grievance reporting, leadership training, and employee stock plan. Compared to a large-scale HB fertiliser company, one plasma-based company had a slightly higher score (6.0) in total, as the plasma-assisted process exhibited the environmental benefits of less CO_2_ emissions and toxic waste generation, and improved water-efficient process using renewable energy sources. However, the large-scale fertiliser company made tremendous efforts on the social pillar, including employee engagement, human rights, and well-being at work. These efforts could reflect a broad trend of corporate social responsibility (CSR) that large companies adopt to align with the expectations from stakeholders and regulations.

To summarise, plasma-technology has high potential to become a sustainable alternative for NH_3_ synthesis in the future. The outcome of E&S analysis provides the direction of improvement of sustainability of performance, suggesting that shareholders should make a significant effort on environmental and social performance. On the other hand, this demonstrated methodology could also give guidance for ESG assessment towards other industries, such as steel and cement manufacturing. In the context of highly polluting industries, it is expected to have major differences in social responsibility, ethical governance and environmental sustainability, since social equity, sustainable development and decarbonisation have become increasingly crucial for long-term development.

As an outlook, our future work on the companies' sustainability (ESG) performances aims to be more comprehensive by also providing an assessment of governance issues, including corporate practice, risk management and stakeholder engagement.

## Data availability

The data underlying this article are available within the article and its online ESI.†

## Conflicts of interest

There are no conflicts to declare.

## Supplementary Material

SU-003-D4SU00423J-s001
