# Supplementary material for: ESG assessment methodology for emerging technologies: plasma versus conventional technology for ammonia production
Source: RSC Sustain. 2024 Dec 12;3(3):1102–13. doi: 10.1039/d4su00423j (PMC11664233; doi:10.1039/d4su00423j)
Supplement: SU-003-D4SU00423J-s001 [file SU-003-D4SU00423J-s001.pdf]

Supplementary Information (SI) for RSC Sustainability.
This journal is © The Royal Society of Chemistry 2024

Supporting Information

ESG Assessment Methodology for Emerging Technologies: Plasma- versus Conventional Technology for Ammonia Production

Le Yu[1,3*,] Amin Keilani[1,2], Nam Nghiep Tran[1,3], Marc Escribà-Gelonch[4] , Michael Goodsite[3] , Sukhbir Sandhu[5] , Harpinder Sanhu[6] , Volker Hessel1[3,7*]

[1] School of Chemical Engineering, Faculty of Science, Engineering and Technology, The University of Adelaide, Adelaide, Australia.

[2] Institute of Process and Particle Engineering, Graz University of Technology, Graz, Austria.

[3] Institute of Sustainability, Energy and Resources (ISER), The University of Adelaide, Adelaide, Australia.

[4] Centre for Workplace Excellence (CWeX), UniSA (University of South Australia) Business, University of South Australia, Adelaide, Australia.

[5] Future Regions Research Centre (FRRC), Federation University Australia, Ballarat, Australia.

[6] School of Engineering, University of Warwick, Coventry, United Kingdom.

1. Environmental pillar

1.1. Risk exposure

Table S1 Risk exposure criteria for environmental pillar

Issues	Criteria	1	2	3	4	5
Carbon footprint	t CO_{2e}/t NH_3	0.018	0.069	N/A	N/A	N/A
Water consumption	water recycling	Minimal water consumption				
Toxic waste	Total solid waste	No toxic waste				
Opp. In renewable energy	Renewable source used	Renewable sources for electricity supply				

Table S2 Risk exposure score of five plasma-technology companies

Issues	Risk Score	1	2	3	4	5
Carbon footprint	2-10	2	3	2	2	2
Water consumption		2	2	2	2	2
Toxic waste		2	2	2	2	2
Opp. In renewable energy		2	2	2	2	2

1.2. Risk management

Table S3 Risk management criteria for carbon footprint issue

Categories	Description	Results	Management score
Mitigation actions	1. Use clear sources of energy	Positive/neutral/Passive	1.3/0.9/0.5
	2. GHG capture plan	Positive/neutral/Passive	1.3/0.9/0.5
	3. Energy management and operational efficiency improvement	Positive/neutral/Passive	1.3/0.9/0.5
	4. Reduction of future energy consumption	Positive/neutral/Passive	1.3/0.9/0.5
Targets	5. Carbon or energy efficiency improvement	Positive/neutral/Passive	1.3/0.9/0.5
	6. Demonstrated track record of achieving target	Positive/neutral/Passive	1.3/0.9/0.5
Performance	7. GHG emission reduction plan	Positive/neutral/Passive	2.2/1.3/0.5
		**Total score**	**10**

Table S4 Risk management score for carbon footprint issue

**Company**	**1**	**2**	**3**	**4**	**5**
**Criteria**			**Score**		
Clear sources of energy	1.3	0.9	1	0.5	0.9
GHG capture plan	0.5	0.5	0.5	0.5	0.5
Energy management and operational efficiency improvement	0.5	0.9	0.9	0.5	0.9
Reduction of future energy consumption	0.9	0.9	0.5	0.5	0.9
Carbon or energy efficiency improvement	0.5	0.5	0.5	0.5	0.5
Demonstrated track record of achieving target	0.5	0.5	0.5	0.5	0.5
GHG emission reduction plan	0.5	0.5	0.5	0.5	0.5
**Total Score**	**4.7**	**4.6**	**4.4**	**3.5**	**4.7**

Table S5 Risk management criteria for water consumption issue

Categories	Description	Results	Management score
Mitigation actions	1. Percentage of water consumption from alternative water sources	Positive/neutral/Passive	1.25/0.9/0.5
	2. Water circulation and recycling rate	Positive/neutral/Passive	1.25/0.9/0.5
	3. Executive body responsible for water management	Positive/neutral/Passive	1.25/0.9/0.5
	4. Water-efficient process to reduce water intensity	Positive/neutral/Passive	1.25/0.9/0.5
Targets	5. Carbon or energy efficiency improvement target	Positive/neutral/Passive	1.25/0.9/0.5
	6. Demonstrated track record of achieving target	Positive/neutral/Passive	1.25/0.9/0.5
	7. Detailed implementation strategy to achieve the target	Positive/neutral/Passive	1.25/0.9/0.5
Performance	8. Water intensity reduction plan	Positive/neutral/Passive	1.25/0.9/0.5
		**Total score**	**10**

Table S6 Risk management score for water consumption issue

**Company**	**1**	**2**	**3**	**4**	**5**
**Criteria**			**Score**		
Percentage of water consumption from alternative water sources	0	0	0	0	0
Water circulation and recycling rate	1	1	0.9	0.9	0.5
Executive body responsible for water management	1	1	0.9	0.5	0.9
Water-efficient process to reduce water intensity	1.2	1	0.9	0.5	0.5
Carbon or energy efficiency improvement target	1.2	1	0.9	1.2	1.2
Demonstrated track record of achieving target	0	0	0	0	0
Detailed implementation strategy to achieve the target	0	0	0	0	0
Water intensity reduction plan	0	0	0	0	0
**Total Score**	**4.4**	**4**	**3.6**	**3.1**	**3.1**

Table S7 Risk management criteria for toxic waste issue

Categories	Description	Results	Management score
Mitigation actions	1. Environmental management system (EMS) setup	Positive/neutral/Passive	2/1.2/0.5
	2. Percentage of sites with HAZWOPER certifications or ISO 14001	Positive/neutral/Passive	2/1.2/0.5
Targets	3. Target for toxic emission reduction	Positive/neutral/Passive	2/1.2/0.5
	4. Demonstrated track record of achieving the target	Positive/neutral/Passive	2/1.2/0.5
	5. Detailed implementation strategy to reduce air emission, water effluents and hazardous waste	Positive/neutral/Passive	2/1.2/0.5
		**Total score**	**10**

Table S8 Risk management score for toxic waste issue

**Company**	**1**	**2**	**3**	**4**	**5**
**Criteria**			**Score**		
Environmental management system (EMS) setup	0.5	0	0.5	0.5	0.5
Percentage of sites with HAZWOPER certifications or ISO 14001	1.2	2	0.5	0.5	0.5
Target for toxic emission reduction	0.5	0.5	0.5	0.5	0.5
Demonstrated track record of achieving the target	0.5	0.5	0.5	0.5	0.5
Detailed implementation strategy to reduce air emission, water effluents and hazardous waste	0.5	0.5	0.5	0.5	0.5
**Total Score**	**3.2**	**3.5**	**2.5**	**2.5**	**2.5**

Table S9 Risk management criteria for opportunities in renewable energy issue

**Categories**	**Description**	**Results**	**Management score**
Strategy	1. Target to increase investment and develop strategies in clean tech	Positive/neutral/Passive	2/1.2/0.5
Initiatives	2. Application of alternative energy including solar, wind, hydrogen, and waste energy	Positive/neutral/Passive	2/1.2/0.5
	3. Optimization of techniques and systems for efficiency improvement	Positive/neutral/Passive	2/1.2/0.5
	4. Control and prevention of pollution including remediation, recycling, and carbon capture	Positive/neutral/Passive	2/1.2/0.5
Performance	5. Revenue derived from clean tech activities	Positive/neutral/Passive	2/1.2/0.5
		**Total score**	**10**

Table S10 Risk management score for opportunities in renewable energy issue

**Company**	**1**	**2**	**3**	**4**	**5**
**Criteria**			**Score**		
Target to increase investment and develop strategies in clean tech	2	1.2	0.5	0.5	0.5
Application of alternative energy including solar, wind, hydrogen, and waste energy	1.2	2	1.2	1.2	1.2
Optimization of techniques and systems for efficiency improvement	1.2	0.5	1.2	0.5	1.2
Control and prevention of pollution including remediation, recycling, and carbon capture	0.5	0.5	1.2	1.2	1.2
Revenue derived from clean tech activities	0	0	0	0	0
**Total Score**	**4.5**	**4.2**	**4.1**	**3.9**	**4.1**

2. Social pillar

2.1. Risk exposure

Table S11 Risk exposure criteria for occupational safety

PSIF Rate*	TRI Rate*	PS Index*	Score
> 2	> 2	> 20	10
1.8	1.8	30	9
1.5	1.5	40	8
1.3	1.3	50	7
1.1	1.1	60	6
0.8	0.8	70	5
0.5	0.5	80	4
0.3	0.3	90	3
< 0.1	< 0.1	100	2

*Note: PSIF: potential severe injury & fatality, TRI: total recordable incidents, PS: process safety.

Table S12 Risk exposure criteria for human capital development

Employee engagement	Score	Human right	Description	Score	Health and Well-being	Score
< 10	10	Stakeholder Engagement	Disengage, neutral, engage	1/0.6/0.3	Health Services, Life Insurance, Pension Coverage	2.5/1.5/0.5
10	9	Application of Human Rights Standard	Disengage, neutral, engage	1/0.6/0.3		
20	8	Assessment of Severity of Human Rights Impacts	Disengage, neutral, engage	1/0.6/0.3	Working Flexibility (hours and location)	2.5/1.5/0.5
30	7	Inclusion of Impact Mitigation Measures	Disengage, neutral, engage	1/0.6/0.3		
40	6	Remediation of Negative Impacts	Disengage, neutral, engage	1/0.6/0.3	Sick Leave and Parental Leave	2.5/1.5/0.5
50	5	Accountability and Transparency	100-10%	0.2-1		
60	4	Percentage of Human Rights Training	10-80%	0.2-1	Health Care Facilities/Subsidies Coverage	2.5/1.5/0.5
70	3	Equity of Employees	50-10%	0.2-1		
> 80	2	Diversity of Employees	80-10%	0.2-1		
			**Total score**	**10**	**Total score**	**10**

Table S13 Risk exposure criteria for product liability

Chemical safety	Description	Score
Possible presence of harmful chemicals in their product portfolio (containing SVHC and SIN*)	Yes/No	2/0.5
Exposure to the pending chemical regulations in the regions in which company operates	Proportion of a company's total sales in each geographic segment	2-8
	**Total score**	**10**

*Note: SVHC: substances of very high concerns, SIN: Substitute it now.

2.2. Risk management

Table S14 Risk management criteria for occupational safety

Categories	Description	Results	Management score
Strategy	1 Group-wide H&S policy has been established	Positive/neutral/Passive	1.25/0.9/0.5
	2 H&S policy has applied to contractors with a regular audit	Positive/neutral/Passive	1.25/0.9/0.5
	3 Percentage of company's H&S system certified to OHSAS 18001 or ISO 45001 (above 20%)	Positive/neutral/Passive	1.25/0.9/0.5
	4 Executive body is responsible for H&S strategy and performance	Positive/neutral/Passive	1.25/0.9/0.5
Targets	5 H&S targets cover target year, reduction (%) and baseline	Positive/neutral/Passive	1.25/0.9/0.5
	6 Implementation strategy to achieve targets	Positive/neutral/Passive	1.25/0.9/0.5
	7 Demonstrated track record of achieving target	Positive/neutral/Passive	1.25/0.9/0.5
Performance	8 H&S metrics include lost time incident rate, TRI rate and Fatalities	Positive/neutral/Passive	1.25/0.9/0.5
		**Total score**	**10**

Table S15 Risk management score for occupational safety

Company	1	2	3	4	5
**Criteria**			**Score**		
Group-wide H&S policy has been established	0.5	0.5	0.5	0.5	0.5
H&S policy has applied to contractors with a regular audit	0.5	0.5	0.5	0.5	0.5
Percentage of company's H&S system certified to OHSAS 18001 or ISO 45001 (above 20%)	0.9	1	0.9	0.5	0.5
Executive body is responsible for H&S strategy and performance	0.9	0.9	0.9	0.5	0.5
H&S targets cover target year, reduction (%) and baseline	0.5	0.5	0.5	0.5	0.5
Implementation strategy to achieve targets	0.5	0.9	0.5	0.5	0.5
Demonstrated track record of achieving target	0.5	0.5	0.5	0.5	0.5
H&S metrics include lost time incident rate, TRI rate and Fatalities	0.5	0.5	0.5	0.5	0.5
**Total Score**	**4.8**	**5.3**	**4.8**	**4**	**4**

Table S16 Risk management criteria for human capital development

Categories	Description	Results	Management score
Strategy	1 Formal grievance reporting or escalation procedures	Positive/neutral/Passive	1.6/1/0.4
	2 Company monitors employee satisfaction on a regular basis	Positive/neutral/Passive	1.6/1/0.4
Targets	3 Company provides employee stock ownership plan (ESOP) or employee stock purchase plan (ESPP)	Positive/neutral/Passive	1.6/1/0.4
	4 Company provides job-specific development training programs	Positive/neutral/Passive	1.6/1/0.4
	5 Company provides leadership training and talent management programs	Positive/neutral/Passive	1.6/1/0.4
Performance	6 Percentage annual employee turnover	10%-80%	2-0.2
		**Total score**	**10**

Table S17 Risk management score for human capital development

**Company**	**1**	**2**	**3**	**4**	**5**
**Criteria**			**Score**		
Formal grievance reporting or escalation procedures	0.4	0.4	0.4	0.4	0.4
Company monitors employee satisfaction on a regular basis	0.4	0.4	0.4	0.4	0.4
Company provides employee stock ownership plan (ESOP) or employee stock purchase plan (ESPP)	0.4	0.4	0.4	0.4	0.4
Company provides job-specific development training programs	1.3	1.3	1	1	1
Company provides leadership training and talent management programs	0.4	0.4	0.4	0.4	0.4
Percentage annual employee turnover	2	2	2	2	2
**Total Score**	**4.9**	**4.9**	**4.6**	**4.6**	**4.6**

Table S18 Risk management criteria for product liability

Categories	Description	Results	Management score
Strategy	1 Phase-out plan has covered the hazardous chemicals and products	Yes/No	2/0
Targets	2 Demonstrated track record of introducing viable alternatives to high-concern substances	Yes/No	2/0
	3 Initiatives to improve consumer awareness on product chemical content	Yes/No	2/0
	4 Company conducts hazard assessments of its substances or products	Yes/No	2/0
	5 Company discloses substance registrations and use	Yes/No	2/0
		**Total score**	**10**

Table S19 Risk management score for product liability

**Company**	**1**	**2**	**3**	**4**	**5**
**Criteria**			**Score**		
Phase-out plan has covered the hazardous chemicals and products	0	0	0	0	0
Demonstrated track record of introducing viable alternatives to high-concern substances	0.5	0.5	0	0	0
Initiatives to improve consumer awareness on product chemical content	1	1	1	1	1
Company conducts hazard assessments of its substances or products	1.5	1.5	1.5	1.5	1.5
Company discloses substance registrations and use	0.5	0.5	0.5	0.5	0.5
**Total Score**	**3.5**	**3.5**	**3**	**3**	**3**
